# Last Clinic of the Course of Professor Dunglison

**Published:** 1845-10

**Authors:** Samuel G. White

**Affiliations:** Georgia


### CLINICAL LECTURES AND REPORTS.

### PHILADELPHIA HOSPITAL.

*Saturday, February* 22, 1845.

LAST CLINIC OF THE COURSE OF PROFESSOR DUNGLISON.

Reported by Dr. Samuel G. White, of Georgia.

At the commencement of the lecture the Professor introduced the patient who had laboured under OBSTRUCTION AND SPASMODIC CONTRACTION OF THE BOWELS, from a narrowing of some portion of the tube.

Under the simple treatment ordered he has perfectly recovered and will be discharged. For some time, it will be necessary to observe caution in diet, to prevent the reaccumulation of fæcal matters, but further than this nothing will be required. The result of this case shows the great importance, in the treatment of disease, of inquiring into the pathological condition, and directing measures for its relief, rather than for that of its expressions.

Several cases of RHEUMATISM, which were under treatment, have also recovered. In each, the sulphate of quinia was freely administered, and to it the principal curative influence is to be ascribed. From the degree of excitement that existed, it became necessary in one case to conjoin topical depletion by cups; and three grains of opium, with eighteen of quinia, were given during the day. In another, as there was less activity, the opiate and quinia were alone demanded, without any depletion. The disease, in some of these cases, had continued for two weeks, but, without exception, the cases yielded to the remedies directed.

Professor Dunglison here repeated that he considered rheumatism to be largely neuropathic in its nature, and that in its treatment the nervous system should be boldly impressed. No article, he thinks, is better adapted for this than the sulphate of quinia, freely given, in combination or not, with opium, according to circumstances. Unless there is very great vascular activity, he believes it never necessary to have recourse to depletory measures.

The Professor here remarked, that two or three cases of PHTHISIS PULMONALIS had recently proceeded to a fatal termination, in which there was very little or no expectoration. It is possible that the matter, after being coughed up, was occasionally swallowed, but undoubtedly very little was secreted. These cases go to prove that patients labouring under this disease do not die, as has been imagined by some, from exhaustion consequent on profuse discharges from the lungs, but rather from constitutional irritation, developed in part by the recuperative action, the powers of the system being exhausted under the efforts to renovate the diseased structures.

The attention of the class was then directed to a case of

ASCITES

dependent on cardiac disease.

The existence of disease of this important organ was evinced in a marked manner in the physiognomy of the patient. When examined, the heart's action was found to be very tumultuous, but the rhythm was principally disturbed. The evidences of effusion into the peritoneal cavity were marked by the distended state of the abdomen, and fluctuation on palpation. Percussion elicited a clear sound over the anterior paries, from the presence of the intestines, which, being lighter, floated on the surface of the fluid; and a dull sound at the sides from the gravitation of the fluid. On changing the position to the side, the clear sound was elicited by percussion of the uppermost side of the abdomen, whilst the dull sound was heard on percussion of the depending part.

The pathology of the case was manifest. In consequence of irregularity of the circulation induced by disease of the heart, hyperæmia of the abdominal vessels occurred, which relieved itself by transudation of the serous portion of the blood. This, in fact, is the immediate cause of most dropsical effusions:—some disease existing in one or more of the solid viscera, and disturbing the equilibrium of the circulation, local hyperæmia occurs, which terminates in effusion.

The prognosis in dropsical cases is modified by its cause. When, as in the present instance, there is serious organic disease, it must always be unfavourable.

In the treatment of ascites the constitution of the individual must be inquired into. If it be robust, or the patient be plethoric, benefit may accrue from the abstraction of blood. But if, as in the case now under consideration, the patient be feeble, and of broken down constitution from bad habits, we must rely on less energetic measures. In such cases, we usually have recourse to medicines that augment the renal and intestinal secretions, or, in other words, to diuretics, and hydragogue cathartics. These abstract from the blood-vessels a portion of their serous contents, give occasion, therefore, to increased absorption, and thus promote the removal of the effused fluid. Accordingly, in this case, the Professor directed a compound of elaterium and gamboge as a cathartic, with one grain each of calomel, digitalis and squill, three times a day as a diuretic. In addition to the action on the kidneys, the revellent and alterative influence of the mercurial may exert a most salutary sorbefacient agency. From the combined action of these remedies, and attention to the general health of the patient, the case may improve somewhat, but so long as the cause remains, there will be perpetual liability to the recurrence of the dropsical accumulation.

The Professor then proceeded to some observations on a case of

ENDOCARDITIS, COMPLICATED WITH VALVULAR DISEASE.

The patient, a male, entered the hospital with high fever and a jerking pulse, which is apt to exist in inflammation of the endocardium. The peculiar expression of countenance, so often observed in cardiac affections, and so marked in the case last presented to the class, is absent in this instance. He has not suffered recently from rheumatism.

It may be repeated here, that patients who have laboured under acute rheumatism are peculiarly liable to various diseases of the heart. The complication of rheumatism with endocarditis, indeed, so often exists, that some have affirmed it to be present in every case of rheumatism. Although such is not the fact, it occurs so frequently that our attention in acute rheumatism should always be directed to the condition of the heart. Should it be involved, it will be indicated by a *bruit de soufflet*, or "bellows sound," which may be produced by simple hyperæmia of the lining membrane—the endocardium—but much more frequently, the Professor thinks, by a narrowing of the cardiac orifices, the consequence of an effusion of plastic lymph. The rasp, file and saw sounds are mere varieties of the bellows sound, and acknowledge a similar mechanism. The functional expressions of endocarditis are usually marked, but the diagnosis is at times very obscure, unless recourse be had to the physical signs, and even then we may have occasionally to doubt.

The Professor here introduced a diagram on the black board to exhibit the situation of the several valves of the heart, the better to elucidate the physical signs indicative of their morbid conditions. He also made a few observations on the sounds of the heart in health. When the ear is placed over the præcordium, two distinct sounds are audible. The *first* is a slow prolonged sound—the second a sharp, short sound, not unlike the lapping of a dog or the clacking of a valve. The former is very compound in character, and its mode of production has been the subject of much disputation. It is now, however, generally admitted, that it is a combination of the sound produced by the rush of the blood through the heart's cavities, the tension of the auriculo-ventricular valves, and the muscular contraction of the organ. Almost all agree, that the second sound results from the sudden fall of the semilunar valves, as their edges are caught by the refluent blood. The Professor is disposed to believe from observations on the living heart in action, as well as from morbid results, that the semilunar valves participate also in the production of the normal first sound.

Succeeding the second sound there is a short period of repose, and then a renewal of the sounds. If the whole time occupied by the sounds and pause be divided into four periods, two of them will be occupied by the first, one by the second sound, and the remaining one by the repose.

As the function of the valves is that of preventing the reflux of the blood, after it has abandoned a cavity, it can readily be comprehended, that certain morbid sounds may be produced by an insufficiency on the part of these valves, permitting the regurgitation of blood through the orifices. The site and time at which these morbid sounds are audible differ, of course, according to the valves involved.

If the insufficiency exists in the mitral valves, the sound will be perceptible over the mammary region, increasing in intensity towards the apex of the heart, and during the contraction or systole of the organ. Sometimes a double or see-saw sound is produced, if vegetations or other morbid deposits exist on the valves. The first sound of the heart will consequently be disturbed in diseases implicating the mitral valves,—but it can be readily seen, that if the auriculo-ventricular opening be narrowed, an abnormous sound in the same region may also accompany the second sound.

Should the semilunar valves of the aorta be involved, the sound of regurgitation will be most distinct over the third rib—the region of the valves—and follow the course of the great vessels; and it will be synchronous with the diastole of the heart. In this case, therefore, the second sound will be deranged—still it will be here again evident, that if the caliber of the arteries be diminished, there may be an abnormous sound over the same region accompanying the first sound.

By attending, therefore, to the situation in which the abnormous sounds are most distinct, and the time of their occurrence, it may be surmised what valves are in fault. This nicety, however, of diagnosis, does not affect the treatment. In the case under consideration from the blowing accompanying the first sound, and from its being most marked near the nipple or apex of the heart, we infer that the mitral valves are implicated.

The causes of valvular disease are various, but the Professor thinks that, in the majority of cases, they are owing to chronic endocarditis. It is very common to find, in old persons, the valves and lining membrane of the aorta coated with plates of ossific or atheromatous matter, which give rise during life to various morbid sounds.

The treatment of active endocarditis consists in the employment of active antiphlogistic means. When, however, the endocarditis has become chronic, or has left only its results, activity may be out of the question. Attention should, then, be directed to the diet, and to other hygienic measures, as moderate exercise, fresh air, &c. Violent muscular efforts, and mental or moral emotions should be avoided; but there is no objection to the proper exercise of the intellectual faculties,—as, unlike the emotions, this can have no effect on the diseased organ. The patient whose case is now being considered, was directed to be placed on the use of the hydrocyanic acid in doses of one drop, with ten drops of the tincture of digitalis, every morning—and strict attention was ordered to the diet, and general health. When old valvular disease alone exists, medicinal agents can be of little use, and reliance is to be placed solely in the recuperative powers,—and on proper hygienic measures to prevent any corporeal or mental excitement.

With this case the Professor concluded the course of clinical lectures. Before dismissing the class, however, he thought it proper to make a few observations on the plan of instruction pursued in this institution, as it appears not to be understood abroad.

Clinical remarks are generally supposed to be made at the bed side of the patient, and most usually they are so. As, however, this would obviously be impracticable where the class is very large, only a few being able to hear or see any thing, another mode has been adopted here. This is, to select such cases as may be considered proper, and present them to the class assembled in the amphitheatre, with appropriate observations. Although there are disadvantages attending this course, it seems best suited for instruction under all circumstances. It would be utterly impossible for a class of 200 clinical students to examine cases for themselves; and if the thing were practicable, whilst it might be an advantage to the student, it would, in many cases, be destructive to the patient, and in all cases intolerable. Each student has thus an opportunity of seeing the patient, and hearing the remarks of the lecturer. Professor Dunglison has adopted the practice of selecting cases that will elucidate the physiological and pathological subjects to which he had directed the attention of the class, during the week, from the chair of Institutes at Jefferson Medical College. His lectures are consequently methodical, and infinitely more instructive than if the cases were taken at random. Being first acquainted with the physiological conditions, the student can best comprehend the pathological aberrations. Pursuing this plan, he has been enabled, in the present course, to treat of a large class of diseases, exhibit numerous pathological specimens, and make appropriate remarks on them. Indeed, in the short space of four months he has lectured on nearly every class of diseases which the practitioner is called upon to treat. Of the number, however, a better idea may be formed from reviewing a schedule.

The course was begun by some general observations on the various means of diagnosticating diseases, as

Functional phenomena,
Physical signs, including
Auscultation, percussion, inspection, palpation, &c.
Hæmatology,
Inspection of the secretions, as the bile, urine, illustrated by specimens of morbid secretions.

Then followed remarks on the importance of attending to morbid anatomy as an aid to diagnosis and therapeutics; but as an aid only. Having next illustrated the diseases of imbibition, especially by pulmonary transudation, as in hæmoptysis, and encephalic transudation, as in hemiplegia, particular diseases were considered. Under diseases of the *alimentary* canal, lectures were delivered on

Mercurial stomatitis,
Pharyngitis,
Ulceration of the pharynx,
Obstruction of the intestines,
Invagination of the intestines,
Ulceration of the intestines and gastrorrhœa, with specimens and pathological remarks on

Ulceration of the pharynx and larynx,
Invagination of the intestines,
Ulceration of the intestines,
Perforation of the intestines,
Tuberculosis of the peritoneum,
Tumour of the peritoneum.

Under diseases of the *respiratory organs*, lectures were given on

Ulceration of the larynx,
Hæmoptysis,
Pleuritis,
Chronic bronchitis,
Bronchorrhœa,
Tuberculosis of the lung,
Gangrene of the lung,
Asthma, and pulmonary emphysema,
Pneumonia and Asphyxia from drowning, with specimens and pathological remarks on

| | |
|---|---|
| Ulceration of the larynx, | Hæmoptysis, |
| Tuberculosis of the lung in its various stages, | Gangrene of the lung, |
| | Pneumonia. |

Under diseases of the *circulatory apparatus*, lectures were given on

| | |
|---|---|
| Pericarditis, | Hemorrhage from the rectum, |
| Endocarditis and endopericarditis, | Communication between the auricles, |

with specimens and pathological remarks on

| | |
|---|---|
| Pericarditis, | Cartilaginous degeneration of the same, |
| Ossification of the aorta, | Patulous foramen ovale in a woman ninety-eight years of age. |
| Vegetations of the aortic valves, | |

Under diseases of the *glandiform* ganglions, lectures were delivered on

| | |
|---|---|
| Atrophy of the spleen, | Splenitis, |

with specimens and pathological remarks on

| | |
|---|---|
| Scrofulous mesenteric ganglionitis, | Atrophy of the spleen, |
| | Splenitis. |

Under diseases of the *glandular* organs, lectures were given on

| | |
|---|---|
| Mercurial ptyalism, | Disorders of the biliary secretion and bilious diseases, |
| Granular disease of the kidney, | Jaundice of the adult, |
| Whisky liver, | " " infant, |
| Cirrhosis of the liver, | Gall stone, |
| Tubercles of the liver, | |

with specimens and pathological remarks on

| | |
|---|---|
| Hypertrophy of the kidney, | Contraction of the urinary bladder, |
| Atrophy of the same, | Whisky liver, |
| Renal apoplexy, | Cirrhosis of the liver, |
| Dislocation of the kidneys, | Tubercles of the liver. |
| Granular disease of the same, | |

Under diseases of the *skin*, lectures were given on

| | |
|---|---|
| Erythema of the scalp, | Impetigo, |
| Induration of the cellular tissue, | Discoloration of the skin from nitrate of silver. |
| Chronic cutaneous diseases in general, | |

Under diseases of the *nervous system*, lectures were given on

| | |
|---|---|
| Hyperæmia of the brain, | Tremors, |
| Ramollissement of the brain, | Chorea, general and partial, |
| Hemiplegia, | Salaam convulsion, |
| Paraplegia, | Epilepsy, |

with specimens and pathological remarks on

Hyperæmia of the brain.

Under diseases of the *reproductive* organs, specimens were exhibited, with pathological remarks on

| | |
|---|---|
| Ovarian cysts, | Recto-vaginal communication, |
| Cysts of the Fallopian tubes, | Open cancer of the uterus, |
| Fibrous tumour of the uterus, | Scirrhus of the uterus. |

On diseases *involving various organs* lectures were delivered on

| | |
|---|---|
| Intermittent fever, | Dropsy, hepatic, |
| Typhoid fever, | " renal, |
| Scarlatina, | Scrophulosis, |
| Rheumatism, acute and subacute, | Cancerous cachexia. |
| Dropsy, cardiac, | |

Professor Dunglison stated, that he could not take leave of the clinical class without expressing to them his thanks, and those of his colleague, Professor Pancoast, for the attention which had been generally paid to the important subjects brought before them. He was likewise greatly indebted to Drs. Curran, Farquharson, Keating and Wilson, resident physicians to his wards, for the anxiety and industry they had exhibited to enable him to bring before the class cases and pathological specimens,—some of them not occurring in his own wards, but in the out-wards of the Hospital. He was glad, that he had been enabled to lay so much instructive matter before them; and stated, that if the clinical information, which he and his colleague had endeavoured to impart, should hereafter tend, in any degree, to smooth the rugged paths of practice, which all are destined to tread, they would consider themselves amply remunerated for all their unpaid exertions.

[In terminating these reports, the writer, having availed himself of the benefits of their clinical instruction, cannot refrain from expressing his highest opinion of the plan pursued by Professor Dunglison and his colleague, Professor Pancoast, connected with their well known ability in selecting cases, fidelity in representing, and skill in the management of disease. To their unremitting attention, untiring zeal, and disinterested exertions for the improvement of the class, its members cordially testify, and for this they merit and receive its most sincere gratitude.

Samuel G. White.]

